# Pyruvate Treatment Restores the Effectiveness of Chemotherapeutic Agents in Human Colon Adenocarcinoma and Pleural Mesothelioma Cells

**DOI:** 10.3390/ijms19113550

**Published:** 2018-11-10

**Authors:** Eleonora Mungo, Loredana Bergandi, Iris Chiara Salaroglio, Sophie Doublier

**Affiliations:** Department of Oncology, University of Torino, Via Santena 5/bis, 10126 Torino, Italy; eleonora.mungo@unito.it (E.M.); irischiara.salaroglio@unito.it (I.C.S.)

**Keywords:** chemoresistance, pyruvate, carbon metabolism, mitochondria respiratory chain, human colon adenocarcinoma cells, human pleural mesothelioma cells

## Abstract

Emerging evidence supports the idea that a dysfunction in cell metabolism could sustain a resistant phenotype in cancer cells. As the success of chemotherapeutic agents is often questioned by the occurrence of multidrug resistance (MDR), a multiple cross-resistance towards different anti-cancer drugs represent a major obstacle to cancer treatment. The present study has clarified the involvement of the carbon metabolites in a more aggressive tumor colon adenocarcinoma phenotype and in a chemoresistant mesothelioma, and the role of pyruvate treatment in the reversion of the potentially related resistance. For the first time, we have shown that human colon adenocarcinoma cells (HT29) and its chemoresistant counterpart (HT29-dx) displayed different carbon metabolism: HT29-dx cells had a higher glucose consumption compared to HT29 cells, whereas human malignant mesothelioma (HMM) cells showed a lower glucose consumption compared to HT29 cells, accompanied by a lower pyruvate production and, consequently, a higher production of lactate. When treated with pyruvate, both HT29-dx and HMM cells exhibited a re-established accumulation of doxorubicin and a lower survival ability, a decreased activity of multidrug resistance protein 1 (MRP1) and a restored mitochondrial respiratory chain function, improving the effectiveness of the chemotherapeutic agents in these resistant cancer cells.

## 1. Introduction

Chemotherapy, one of the most efficient therapeutic approaches to solid and hematologic cancers, has led to improved remission and survival in many malignancies. Colorectal cancer (CRC) is the third most common type of cancer and has the third highest rate of cancer-associated mortality worldwide [1], whereas malignant mesothelioma (MM) is a highly aggressive and constitutive resistant tumor of the serosal cavities that exhibits a strong correlation with exposure to asbestos fibers [2]. 

Unfortunately, in a significant number of patients, the tumor is not responsive to the therapeutic agents, thus the onset of multidrug resistance (MDR), a multiple cross-resistance towards different anticancer drugs, affects about the 40–70% of tumors at diagnosis [3,4].

One of the main mechanisms of MDR is the overexpression of the ATP-binding cassette (ABC) transporters, such as P-glycoprotein (Pgp) and multidrug related proteins (MRPs), that extrude anticancer drugs, leading to a decrease of the accumulation of intracellular chemotherapeutics and of their cytotoxic effects [5]. Other mechanisms include activation of detoxifying systems, changes in the expression/function of the molecular targets of anticancer drugs, enhanced ability of cancer cells to repair anticancer drug-induced DNA damage, altered expression/function of pro- or anti-apoptotic factors, and enhanced stress responses [6]. Moreover, uncontrolled cell proliferation, the essence of neoplastic disease, involves not only a deregulated control of cell cycle, but also several changes in energy metabolism to fuel cell growth and division [6]. Under aerobic conditions, non-tumoral cells process glucose, first to pyruvate *via* glycolysis in the cytosol and thereafter to carbon dioxide in the mitochondria. Differently, cancer cells reprogram their glucose metabolism limiting their energy metabolism largely to increased glycolysis, known as the Warburg effect, which generally facilitates metastasis and inhibits apoptosis [6,7,8,9]. 

Emerging evidence supports the idea that the deregulated cell metabolism could also sustain drug resistance [10,11].

In the present study, we clarified the role of the carbon metabolism in the development of a more aggressive tumor colon adenocarcinoma and in the malignant mesothelioma phenotype. Moreover, we have investigated whether pyruvate treatment may restore the cytotoxic effects of chemotherapeutic agents in drug-resistant cells.

## 2. Results

### 2.1. Human Colon Adenocarcinoma Cells (HT29), HT29-dx and Human Malignant Mesothelioma Cells (HMM) Had a Different Carbon Metabolism 

**Figure 1:**
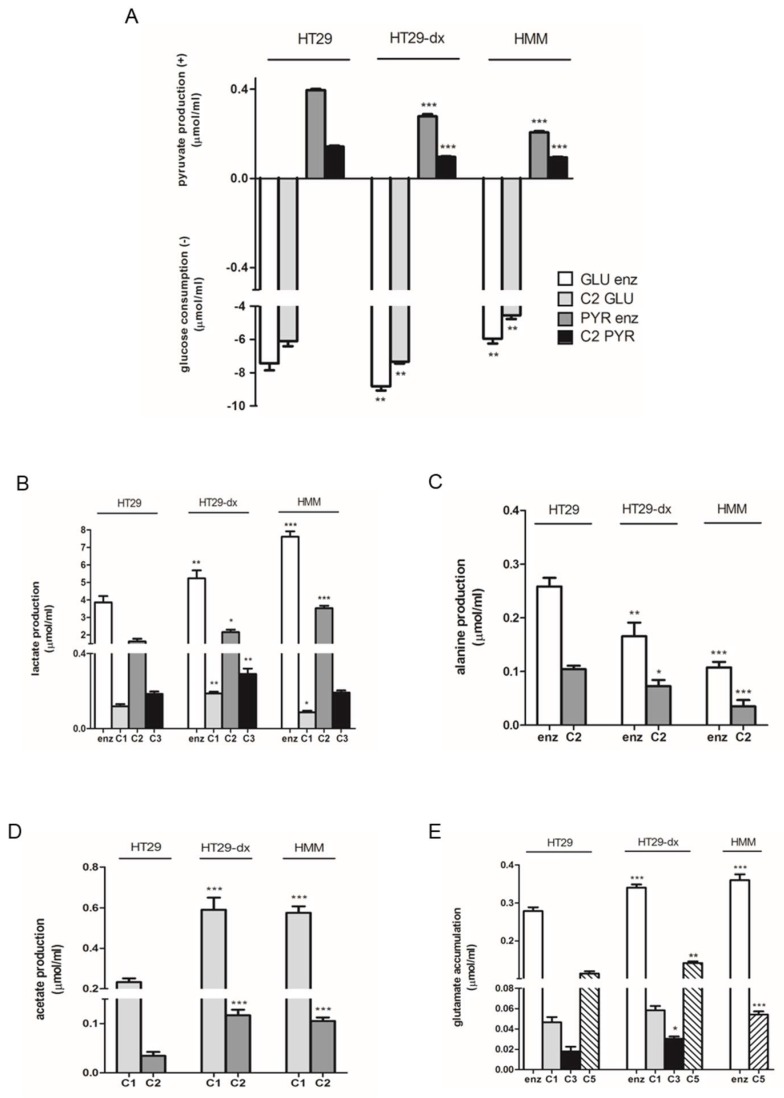
Carbon metabolism in HT29, HT29-dx and HMM cancer cells: (**A**) glucose consumption (−) and pyruvate production (+); (**B**) lactate production; (**C**) alanine production; (**D**) acetate production; and (**E**) glutamate accumulation. Results in quadruplicate, given as µmol/mL, are presented as means ± SEM (*n* = 4). Each enzymatically and ^13^C NMR measurements versus HT29: * *p* < 0.01; ** *p* < 0.001; *** *p* < 0.0001. (**A**) GLU Enz., glucose measured enzymatically; C2 GLU, 2-^13^C-glucose measured by NMR; PYR Enz., pyruvate measured enzymatically; C2 PYR, 2-^13^C-pyruvate measured by NMR. (**B**–**E**) Enz., lactate, alanine, acetate and glutamate measured enzymatically; C1, C2, C3 and C5 GLU, measured by ^13^C NMR.

To investigate the energetic metabolism of glucose, we measured different metabolites by the enzymatic methods and ^13^C NMR technique in HT29, in their chemoresistant counterpart HT29-dx cells and in HMM (Figure 1). 

We observed that HT29-dx cells had a higher glucose consumption compared to HT29 cells, whereas HMM cells showed a lower glucose consumption compared to HT29 cells, even though glucose was consumed with avidity by all the cell types (Figure 1A). Consequently, the pyruvate level increased in all the cell lines during the incubation time (as described in Section 4), and we observed that the production of pyruvate was significantly lower in HT29-dx and HMM cells compared to HT29 cells (Figure 1A). Moreover, as shown by both techniques, HT29-dx and HMM cells produced a higher amount of lactate compared to HT29 cells (Figure 1B).

In fact, the 2-^13^C-lactate, derived from 2-^13^C-pyruvate by lactate dehydrogenase (LDH), represented about the 31.7%, 35.9% and 83.3% of consumed glucose in HT29, HT29-dx and HMM cells, respectively, without any increase in ^13^CO_2_ production in HT29-dx (47.5%) and a significant decrease in ^13^CO_2_ production in HMM cells (11.8%) compared to HT29 cells (55.1%). These data suggest that the fate of glucose carbon 2 was very different in HT29-dx and HMM cells (Figure S1A). Moreover the decrease in 1-^13^C-lactate synthesis in HMM cells was also consistent with a decrease in Krebs cycle performance accompanied not only by a significant decrease in ^13^CO_2_ production, but also by a reduced mitochondrial functioning measured as a dramatic decrease in intramitochondrial reduced nicotinamide adenine dinucleotide (NADH) transport in these cells (10.9 ± 1 µmol/mL in HT29, 12.33 ± 0.66 µmol/mL in HT29-dx and 4.25 ± 0.35 µmol/mL in HMM (*p* < 0.001)) (Figure S1B).

The total amount of the lactate labeling in C1, C2 and C3 was approximately equal to half of the formed lactate when measured enzymatically, indicating that in HT29, HT29-dx and HMM cells the lactate production originated from the consumed glucose (Figure 1B). In addition, the labeling of lactate on its carbon 3 showed that lactate was re-synthesized through a futile cycle, after which the 2-^13^C-pyruvate was formed through the consequent activity of the pyruvate carboxylase (PC) enzyme to form 2-^13^C-oxaloacetate and of the phosphoenolpyruvate carboxykinase (PCK2) enzyme to form phosphoenolpyruvate. Moreover, with a whole Krebs cycle, 2-^13^C-pyruvate was converted into 3-^13^C-citrate, 2- and 3-^13^C-succinate, and 2- and then 3-^13^C-malate, preceding the formation of 2- and 3-^13^C-pyruvate by the malic enzyme through another futile cycle. The labeling of lactate on its carbon 1 showed that lactate has also been indirectly synthesized not only by PC, but also by pyruvate dehydrogenase (PDC) enzyme, leading to the synthesis of 1-^13^C-oxaloacetate.

In accordance with the decrease in pyruvate and the increase in lactate production in both HT29-dx and HMM cells, the alanine production, measured enzymatically and through the labeling of C2, was lower compared to HT29 cells (Figure 1C).

The measurement of the acetate level was not possible enzymatically, but the labeling of C1 and C2 showed a higher acetate production, derived from acetyl-CoA, as a product of glucose metabolism, for action of a hydrolase (EC 3.1.2.1) enzyme in HT29-dx and HMM cells compared to HT29 cells (Figure 1D). Such acetate accumulation revealed that citrate synthase enzyme, which initiates the Krebs cycle combining one molecule of oxaloacetate with one molecule of acetyl-CoA, is a limiting step in these two cell types.

As shown in Figure 1E, HT29-dx and HMM cells accumulated more glutamate compared to HT29 cells, indicating that both cell lines had a greater production of ^13^C-glutamate that derived from 2-^13^C-glucose consumption and, consequently, from the Krebs cycle through the action of glutamate dehydrogenase enzyme on alpha-ketoglutarate molecules that derived from acetyl-CoA (5-^13^C-alpha-ketoglutarate) and from oxalacetate (3-^13^C-alpha-ketoglutarate).

The lack of the increase in ^13^CO_2_ production in HT29-dx compared to HT29 cells suggests a limitation of the functioning of the Krebs cycle, which did not respond to an increase in pyruvate levels and afterwards acetyl-CoA production. Indeed, acetyl-CoA was more converted into acetate than citrate and the glucose carbon 2 was slightly incorporated into carbons 3 and 5 of glutamate. Otherwise, the fate of glucose carbon 2 in HMM cells was very different compared to HT29 cells. The increased 2-^13^C-lactate production in HMM cells was accompanied by a significant decrease both in ^13^CO_2_ production and in 1-^13^C-lactate synthesis, derived by an indirect synthesis through futile cycles, suggesting a reduction in Krebs cycle.

**Figure 2:**
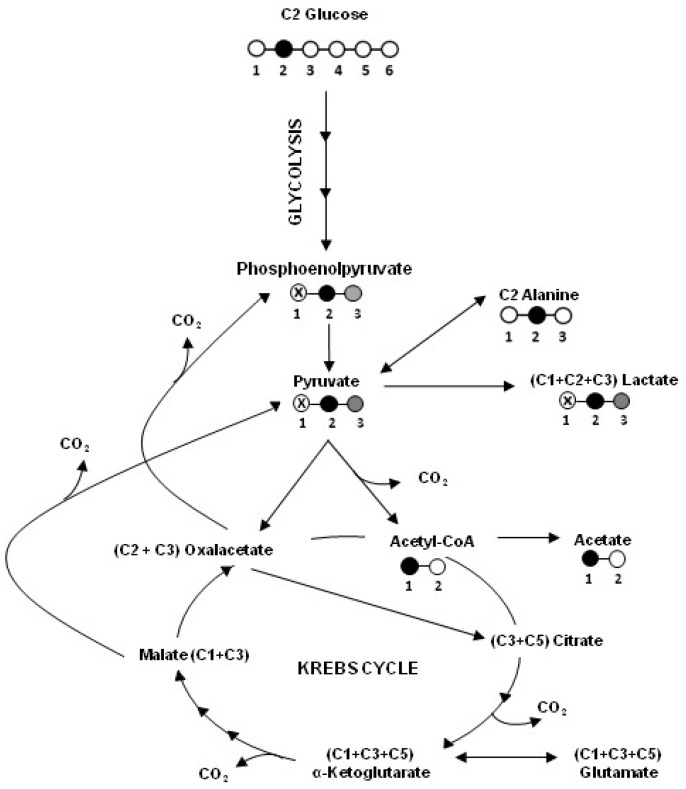
A schematic representation of the ^13^C-labeling patterns of the main metabolites formed from 2-^13^C-glucose in HT29, HT29-dx and HMM cells.

Figure 2 shows a schematic representation of the ^13^C-labeling patterns of the main metabolites formed from 2-^13^C-glucose in HT29, HT29-dx and HMM cells.

### 2.2. Phosphoenolpyruvate Carboxykinase 2 (PCK2), Lactate Dehydrogenase (LDH-A) mRNA Expression and LDH Activity Were Higher in HT29-dx Cells

We analyzed the mRNA expression of enzymes involved in glycolysis and gluconeogenesis using a PrimerPCR Pathway Plate.

**Figure 3:**
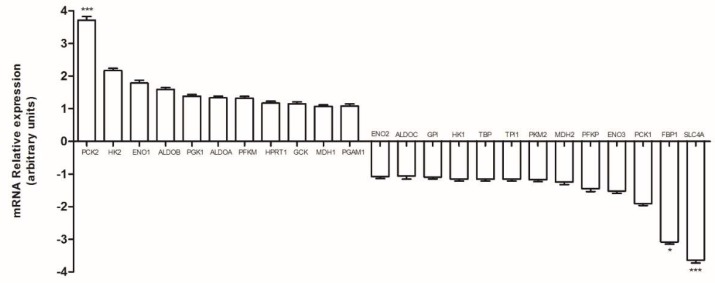
Polymerase chain reaction (PCR) array analysis of glycolysis and gluconeogenesis enzymes in HT29-dx cells. The levels of messenger RNA (mRNA), expressed as relative fold change, from HT29 and HT29-dx cells was analyzed by PCR array specific for glycolysis and gluconeogenesis related genes, as reported under Section 4. Versus HT29: * *p* < 0.1 and *** *p* < 0.0001. PCK2: phosphoenolpyruvate carboxykinase 2; HK2: hexokinase 2; ENO1: enolase 1; ALDOB: aldolase B; PGK1: phosphoglycerate kinase 1; ALDOA: aldolase A; PFKM: phosphofructokinase; HPRT1: hypoxanthine phosphoribosyltransferase 1; GCK: glucokinase; MDH1: malate dehydrogenase 1; PGAM1: phosphoglycerate mutase 1; ENO2: enolase 2; ALDOC: aldolase C; GPI: glucose-6-phosphate isomerase; HK1: hexokinase 1; TBP: TATA binding protein; TPI1: triosephosphate isomerase 1; PKM2: pyruvate kinase 2; MDH2: malate dehydrogenase 2; PFKP: phosphofructokinase; ENO3: enolase 3; PCK1: phosphoenelpyruvate carboxykinase 1; FBP1: fructose-1,6-bisphosphatase 1; SLC4A: solute carrier family 2 member 4.

Among all the investigated genes (Figure 3), only the solute carrier family 2 member 4 (SLC2A4) and the fructose-1,6-bisphosphatase 1 (FBP1), an enzyme of gluconeogenesis, were downregulated, whereas *PCK2* gene was upregulated in HT29-dx cells compared to HT29 cells. The increased PCK2 mRNA level, which catalyzes the conversion of oxaloacetate to phosphoenolpyruvate in the presence of guanosine-5′-triphosphate (GTP), was in agreement with the metabolomic data in HT29-dx cells. Indeed, the labeling of the increased carbon 3 lactate production suggested that part of lactate was synthesized from the pyruvate through the PC and the PCK2 actions.

**Figure 4:**
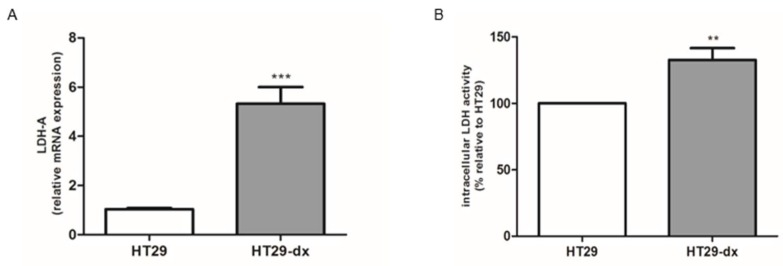
Lactate dehydrogenase isoform A (LDH-A) mRNA expression and LDH activity in HT29 and HT29-dx cells. (**A**) The expression of LDH-A gene was normalized to β-2 microglobulin as endogenous control gene. LDH-A mRNA level, expressed as relative fold change, was analyzed by quantitative real-time polymerase chain reaction (RT-qPCR, *n* = 6). Versus HT29: *** *p* < 0.0001. (**B**) LDH intracellular activity was measured spectrophotometrically in duplicate (*n* = 4), as described under Section 4. The results were expressed as intracellular LDH activity. Versus HT29 cells: ** *p* < 0.001.

It is well known that LDH-A is involved in tumor initiation and cancer metabolism (fermentative glycolysis) resulting in increased lactate production, even under oxygen-sufficient conditions (a process known as the Warburg effect) [12]. As we demonstrated that HT29-dx cells had a higher production of lactate and a lower production of pyruvate, we focused our attention on the expression of LDH-A and on LDH activity that catalyzes the interconversion of pyruvate to lactate. The LDH-A mRNA expression and the LDH intracellular activity were significantly increased in HT29-dx cells compared to HT29 cells (Figure 4).

### 2.3. Pyruvate Restored the Sensibility to Doxorubicin in Both HT29-dx and HMM Cells, Modifying the Multidrug Resistance Protein 1 (MRP1) Activity

**Figure 5:**
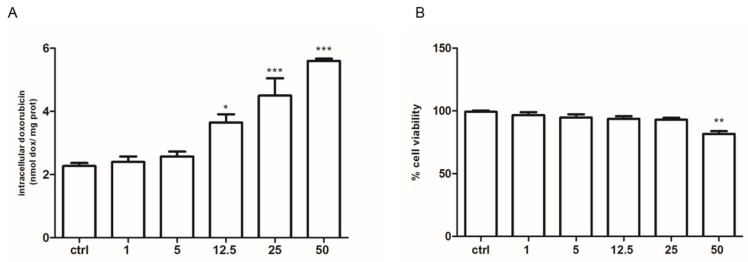
Effect of pyruvate on doxorubicin accumulation and on cell viability using a neutral red uptake assay in HT29-dx cells. (**A**) For doxorubicin accumulation measurement, HT29-dx cells were incubated in fresh medium, with 5 µM doxorubicin for 24 h in the absence (ctrl) or in the presence of pyruvate at 1, 5, 12.5, 25 and 50 mM. Then, the intracellular drug content was detected fluorometrically in duplicate (see Section 4 for details). The experiments were performed in triplicate and data are represented as mean ± SEM (*n* = 6). Versus ctrl: * *p* < 0.1; *** *p* < 0.0001. (**B**) For cell viability assay, HT29 dx cells were incubated in the absence (ctrl) or in the presence of pyruvate at 1, 5, 12.5, 25 and 50 mM. The plates were then incubated for 1 h at 37 °C with a medium containing neutral red solution. The dye was extracted in each well and the absorbance was read at 540 nm using a spectrophotometer. The absorbance was expressed in percent of cell viability versus control (ctrl). The experiments were performed in triplicate and data are represented as mean ± SEM (*n* = 3). Versus ctrl ** *p* < 0.01.

**Figure 6:**
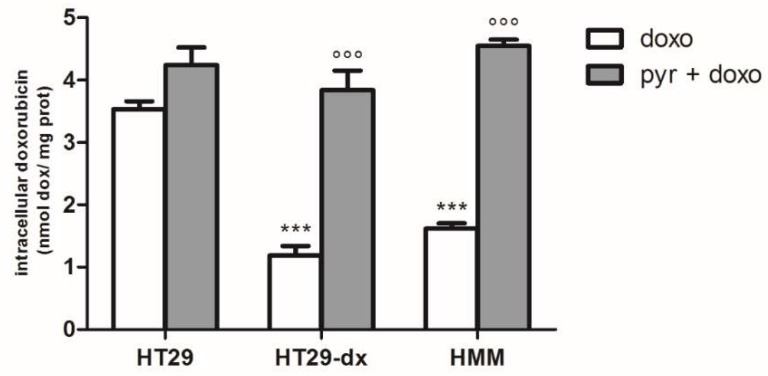
Effect of pyruvate on doxorubicin in HT29, HT29-dx and HMM cells. The cells were incubated in fresh medium containing 5 µM doxorubicin (doxo) in the absence or in the presence of 25 mM pyruvate (pyr) for 24 h. Then, the intracellular drug content was detected fluorometrically in duplicate (see Section 4 for details). The experiments were performed in triplicate and data are represented as mean ± SEM (*n* = 6). HT29-dx cells in the presence of doxo versus HT29 cells in the presence of doxo *** *p* < 0.0001; HMM cells in the presence of doxo versus HT29 cells in the presence of doxo *** *p* < 0.0001; HT29-dx cells in the presence of pyr and doxo versus HT29-dx cells in the presence of doxo °°° *p* < 0.0001; HMM cells in the presence of pyr and doxo versus HMM cells in the presence of doxo °°° *p* < 0.0001.

We observed that after 24 h incubation with 5 µM doxorubicin the cellular accumulation of doxorubicin was significantly reduced in HT29-dx cells compared to HT29 cells, confirming what was previously described by Riganti et al. [13]. Knowing that HT29-dx cells had a lower production of pyruvate, we evaluated the effect of the pyruvate treatment on intracellular doxorubicin accumulation. Therefore, based on a previous study regarding the effect of pyruvate treatment on cells derived from patients with mitochondrial diseases [14], we incubated HT29-dx cells with increasing doses of pyruvate (1–50 mM range) and we observed that the cells accumulated more doxorubicin significantly starting from 12.5 mM pyruvate, but with a more evident effect from 25 mM (Figure 5). Finally, 25 mM of pyruvate was chosen to perform all the experiments as, at this concentration, it was no cytotoxic, and it was able to significantly revert doxorubicin accumulation in HT29-dx. Similar results were obtained in the chemoresistant HMM cells (Figure 6).

To confirm the obtained results, we analyzed the activity of ATP binding cassette transport proteins in the absence and in the presence of pyruvate, using the assay of rhodamine 123.

**Figure 7:**
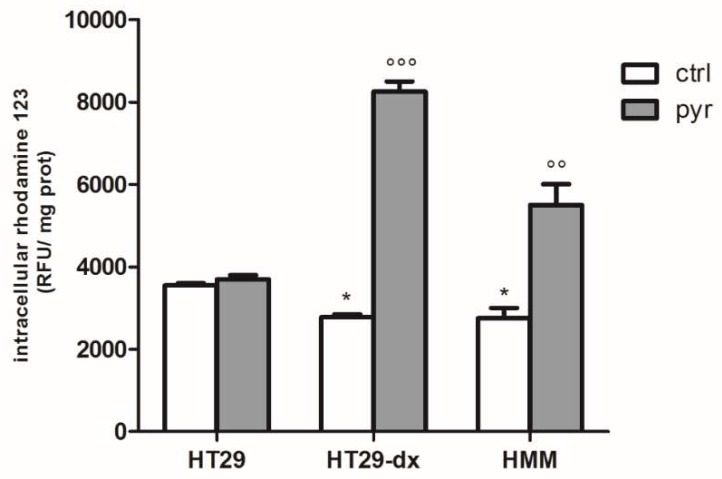
ATP binding cassette transporter activity using the assay of rhodamine 123. The cells were incubated in fresh medium in the absence or in the presence of pyruvate (pyr) 25 mM for 24 h. Rhodamine 123 (1 ng/mL) was added to each sample and intracellular fluorescence of rhodamine 123, expressed as relative fluorescence units (RFU/mg) cell proteins, was detected fluorometrically in duplicate, using a LS-5 spectrofluorometer (PerkinElmer, Waltham, MA, USA). The experiments were performed in triplicate and data are represented as mean ± SEM (*n* = 6). HT29-dx cells versus HT29 cells * *p* < 0.01; HMM cells versus HT29 cells * *p* < 0.01; HT29-dx cells in the presence of pyr versus HT29-dx cells in the absence of pyr (ctrl) °°° *p* < 0.0001; HMM cells in the presence of pyr versus HMM cells in the absence of pyr (ctrl) °° *p* < 0.001.

**Figure 8:**
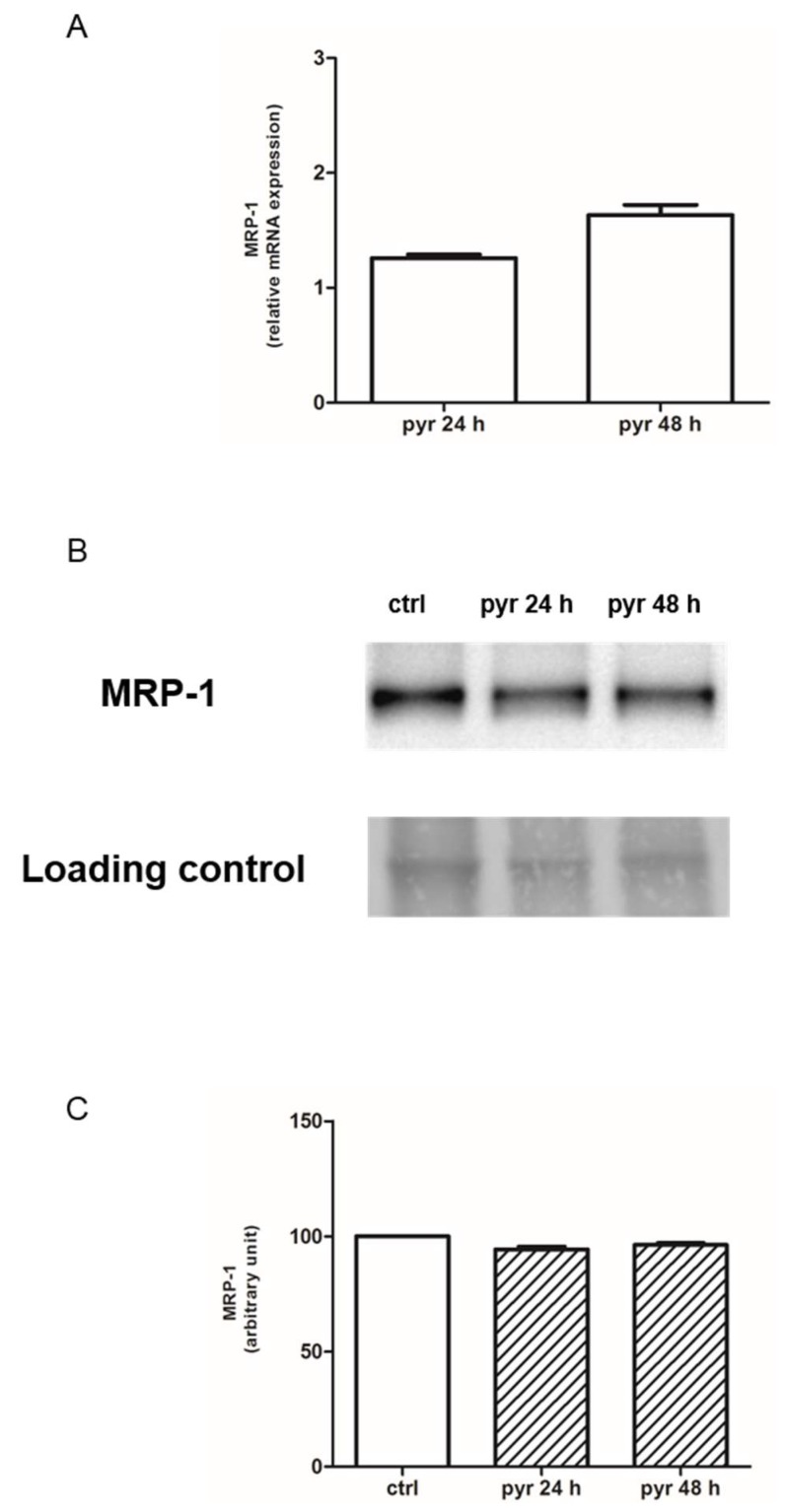
MRP1 mRNA expression in HT29-dx cells tested by qRT-PCR and Western blotting analysis in absence or in the presence of pyruvate. (**A**) HT29-dx cells were incubated in fresh medium in the absence or in the presence of pyruvate 25 mM for 24 and 48 h. The expression of MRP1 was normalized to β-2 microglobulin as endogenous control gene. Levels of mRNA, expressed as relative fold change, of MRP1 gene analyzed by quantitative real-time polymerase chain reaction (RT-qPCR, *n* = 6). (**B**) Western blotting detection of MRP1 protein in HT29-dx cells. The protein membrane extracts (45 µg) obtained using the Pierce Cell Surface Protein Isolation Kit (Pierce Biotechnology, Thermo Scientific, Waltham, USA) were separated by SDS-PAGE, transferred to polyvinylidene difluoride (PVDF) membrane and blotted with anti-MRP1 antibody. Ponceau was used as loading control for equal protein/well. The figure is representative of three similar experiments. (**C**) The protein bands of three independent experiments were quantified by densitometry and the values are expressed as arbitrary units.

The intracellular fluorescence of rhodamine 123, used as index of Pgp plus MRP1 activity, was significantly lower in HT29-dx and HMM cells compared to HT29 cells, whereas it was significantly higher in HT29-dx and HMM cells treated with pyruvate compared to the untreated ones (Figure 7). As MRP1 expression was significantly higher in HT29-dx [15], we assessed the expression of MRP1 in HT29-dx cells after pyruvate treatment. As shown in Figure 8A, we observed that MRP1 mRNA expression in HT29-dx was similar in the absence or in the presence of pyruvate (both after 24 and 48 h incubation). Similarly, Western blot analysis showed that MRP1 protein levels were equal in control cells and after treatment with pyruvate (Figure 8B,C). These results suggest that pyruvate was able to revert the resistance to doxorubicin in HT29-dx cells lowering the MRP1 activity. Although we lack a mechanistic explanation of the impaired MRP1 activity after pyruvate treatment, without any change in gene and protein expression, the results strongly demonstrated a restoration in intracellular doxorubicin accumulation in chemoresistant cells after pyruvate incubation.

### 2.4. Sodium Oxamate and UK5099 Partially Concurred to the Effect of Pyruvate in Restore the Sensibility to Doxorubicin in HT29-dx and HMM

**Figure 9:**
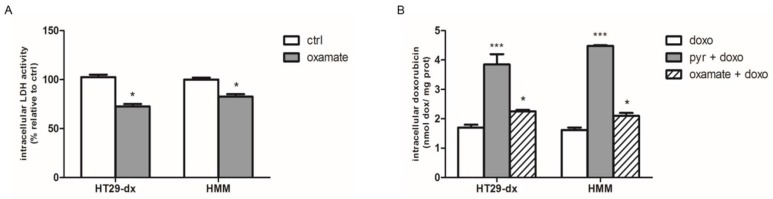
Effect of oxamate on LDH activity and on doxorubicin accumulation in HT29-dx and HMM cells. (**A**) The cells were incubated in fresh medium in the absence (ctrl) or in the presence of 75 mM oxamate for 24 h. LDH intracellular activity was measured spectrophotometrically in duplicate (*n* = 4), as described under Section 4. HT29-dx cells in the presence of oxamate versus HT29-dx ctrl * *p* < 0.01; HMM cells in the presence of oxamate versus HMM ctrl * *p* < 0.01. (**B**) The cells were incubated in fresh medium containing 5 µM doxorubicin (doxo) in the absence or in the presence of 25 mM pyruvate (pyr) or in the presence of 75 mM oxamate for 24 h. Then, the intracellular drug content was detected fluorometrically in duplicate (see Section 4 for details). The experiments were performed in triplicate and data are represented as mean ± SEM (*n* = 6). HT29-dx cells in the presence of pyr and doxo versus HT29-dx cells in the presence of doxo *** *p* < 0.0001; HT29-dx cells in the presence of oxamate and doxo versus HT29-dx cells in the presence of doxo * *p* < 0.01; HMM cells in the presence of pyr and doxo versus HMM cells in the presence of doxo *** *p* < 0.0001; HMM cells in the presence of oxamate and doxo versus HMM cells in the presence of doxo * *p* < 0.01.

The cells were treated with 50, 75 and 100 mM sodium oxamate for 24–48 h, and then intracellular LDH activity was measured to confirm the potential LDH-A inhibition by oxamate in HT29-dx and HMM cells. As shown in Figure 9A, we found that 75 mM oxamate inhibited about 24% of LDH activity at 24 h, while oxamate at 75 mM for 48 h and 100 mM for 24 h exerted higher toxicity so that it was not possible stimulate cells with doxorubicin. Considering the partial LDH inhibition, we observed that after 75 mM oxamate the resistant cells accumulated a small amount of doxorubicin (Figure 9B). 

**Figure 10:**
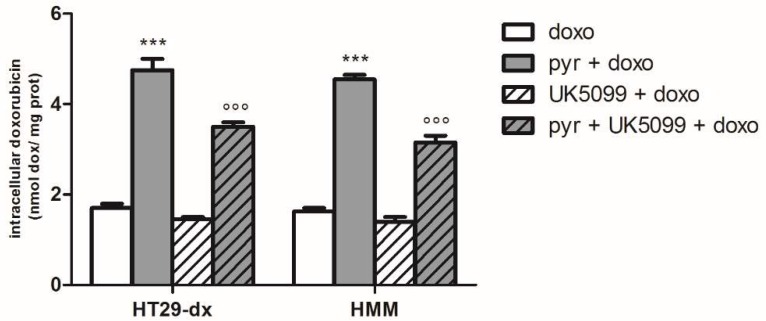
Effect of pyruvate and mitochondrial pyruvate carrier inhibitor (UK5099) on doxorubicin accumulation in HT29-dx and HMM cells. The cells were incubated in fresh medium containing 5 µM doxorubicin (doxo) for 6 h in the absence or in the presence of 25 mM pyruvate (pyr) or in the presence of 20 µM UK5099 or 25 mM pyruvate (pyr) and 20 µM UK5099 for 48 h. Then, the intracellular drug content was detected fluorometrically in duplicate (see Section 4 for details). The experiments were performed in triplicate and data are represented as mean ± SEM (*n* = 6). HT29-dx cells in the presence of pyr and doxo versus HT29-dx cells in the presence of doxo *** *p* < 0.0001; HMM cells in the presence of pyr and doxo versus HMM cells in the presence of doxo *** *p* < 0.0001; HT29-dx in the presence of pyr, UK5099 and doxo versus HT29-dx cells in the presence of pyr and doxo °°° *p* < 0.0001; HMM in the presence of pyr, UK5099 and doxo versus HMM cells in the presence of pyr and doxo °°° *p* < 0.0001.

**Figure 11:**
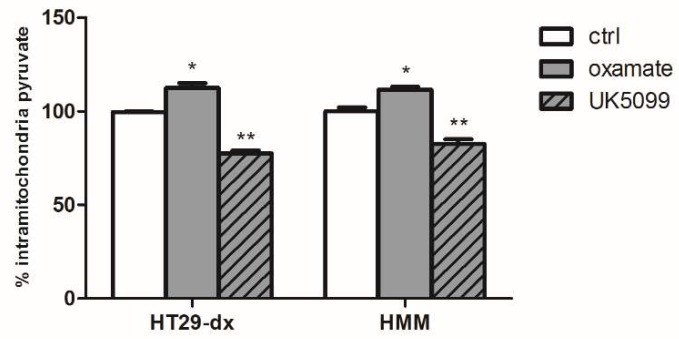
Effect of the LDH inhibitor (oxamate) and the MPC blockade with UK5099 on intramitochondrial pyruvate concentration in HT29-dx and HMM cells. The cells were incubated in fresh medium containing 25 mM pyruvate (ctrl) in the presence of 75 mM oxamate for 24 h or 20 µM UK5099 for 48 h. The experiments were performed in duplicate and data are represented as mean ± SEM (*n* = 4). HT29-dx and HMM cells in the presence of oxamate versus HT29-dx and HMM cells ctrl * *p* < 0.01; HT29-dx and HMM cells in the presence of UK5099 versus HT29-dx and HMM cells ctrl ** *p* < 0.001.

To evaluate whether mitochondrial pyruvate was specifically required for the observed phenotype, the UK5099, a mitochondria pyruvate carrier (MPC) inhibitor, was used. As shown in Figure 10, 20 μM UK5099 for 48 h promoted a significant reduction in doxorubicin accumulation induced by pyruvate in HT29-dx and HMM cells. Despite the reversion of sensitive phenotype after pyruvate treatment, demonstrating that intramitochondrial pyruvate is required for this phenomenon, the lack of complete effect of UK5099 in doxorubicin accumulation is probably due to a significantly, but not total, reduced pyruvate concentration in mitochondria, as reported by Zhong Y. et al. and Li X. et al. [16,17]. Indeed, there was significantly reduced intramitochondrial pyruvate levels after UK5099 incubation for 48 h in HT29-dx and HMM cells (Figure 11). Moreover, as shown in Figure 11, the LDH-A inhibition contributed to increase the mitochondrial pyruvate concentration. Thus, when the LDH is inhibited, even if partially, pyruvate enters the TCA cycle, contributing to restore doxorubicin accumulation in chemoresistant cells. 

### 2.5. Pyruvate Restored Drugs Cytotoxicity in Both HT29-dx and HMM Cells

We then analyzed the potential reversion of the cytotoxic effect, evaluated as inhibition of survival ability, of doxorubicin and of other drugs that were substrates of MRP1, such as 5-fluorouracil and oxaliplatin.

**Figure 12:**
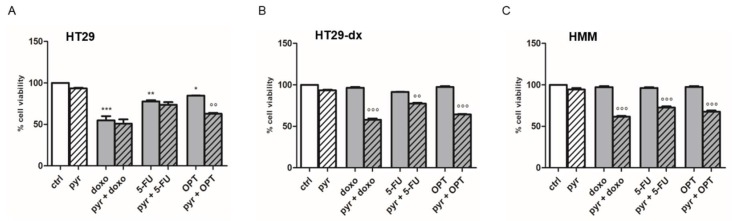
Effect of pyruvate on cell viability using a neutral red uptake assay in HT29, HT29-dx and HMM cells. (**A**) HT29; (**B**) HT29-dx; and (**C**) HMM cells were seeded in 96-well tissue culture plates (25,000 cells/well). After 24 h, the cells were incubated in fresh medium with pyruvate 25 mM containing 5 μM doxorubicin or 150 μM of 5-FU or 30 mM of OPT for 48 h. The plates were then incubated for 1 h at 37 °C with a medium containing neutral red solution. The dye was extracted in each well and the absorbance was read at 540 nm using a spectrophotometer. The absorbance was expressed in percent of cell viability versus control (ctrl). The experiments were performed in triplicate and data are represented as mean ± SEM (*n* = 3). Versus each ctrl * *p* < 0.01; ** *p* < 0.001; *** *p* < 0.0001; versus each drug in absence of pyr °° *p* < 0.001; °°° *p* < 0.0001.

**Figure 13:**
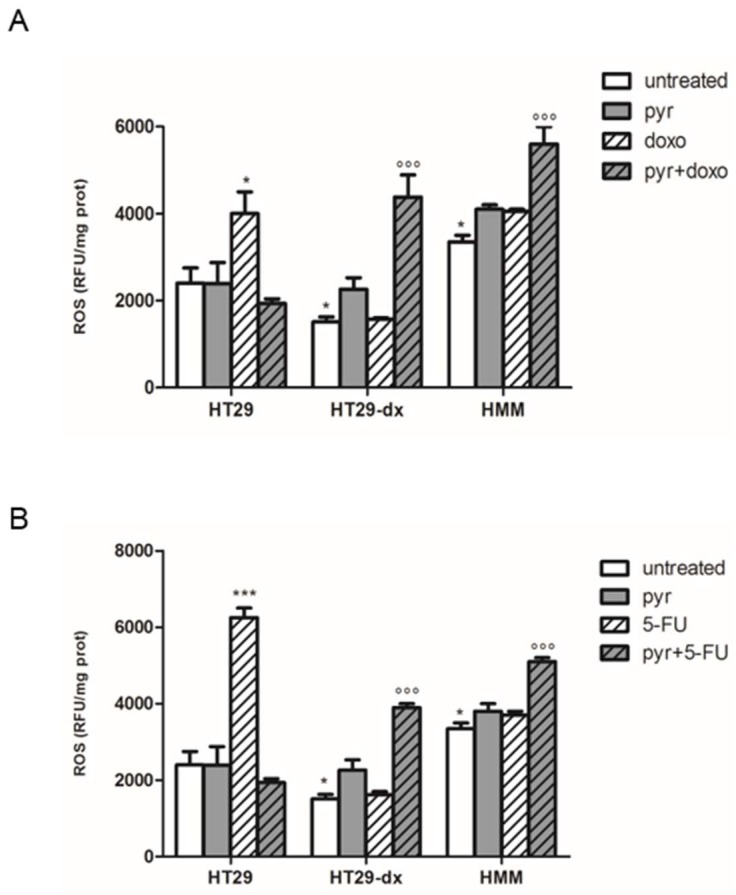

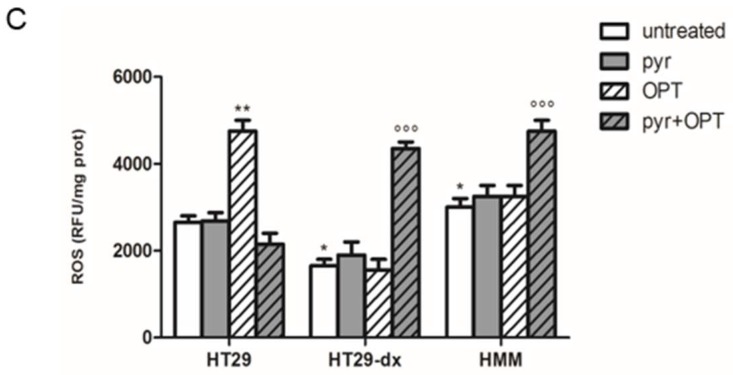
ROS production in the presence of doxorubicin and pyruvate in HT29, HT29-dx and HMM cells. The cells were incubated in fresh medium in the absence (untreated) or in the presence of: pyruvate (pyr) 25 mM, 5 μM doxorubicin (doxo) and pyruvate plus doxorubicin for 24 h (**A**); 150 μM of 5-fluorouracile (5-FU) and pyruvate plus 5-FU for 48 h (**B**); or 30 mM of OPT and pyruvate plus OPT for 48 h (**C**). Then, cells were incubated with 10 μmol/L of the fluorescent probe (DCFDA) for 30 min at 37 °C. The fluorescence of each sample, an indicator of ROS levels, was read at 504 nm (λ excitation) and 529 nm (λ emission). The results were expressed as RFU/mg cell proteins. HT29 cells in the presence of doxo/5-FU/OPT versus HT29 untreated cells * *p* < 0.01; HT29-dx untreated cells versus HT29 untreated cells * *p* < 0.01; HT29-dx cells in the presence of pyr plus doxo/5-FU/OPT versus HT29-dx cells in the presence of doxo/5-FU/OPT °°° *p* < 0.0001; HMM cells untreated cells versus HT29 untreated cells * *p* < 0.01; HMM cells in the presence of pyr plus doxo/5-FU/OPT versus HMM cells in the presence of doxo/5-FU/OPT °°° *p* < 0.0001.

As shown in Figure 12, doxorubicin (doxo), 5-fluorouracil (5-FU) and oxaliplatin (OPT) had a maximal cytotoxic effect in HT29 cells after 48 h of treatment, whereas the three drugs alone had no effect in HT29-dx and HMM cells. Moreover, the addition of pyruvate did not modify cell viability in HT29 cells (Figure 12A). Differently, in HT29-dx (Figure 12B) and HMM (Figure 12C) cells, pyruvate diminished cell viability when added to doxorubicin, 5-fluorouracil or oxaliplatin. As expected, doxorubicin, 5-fluorouracil and oxaliplatin increased the reactive oxygen species (ROS) production in HT29 cells, but not in HT29-dx and HMM cells (Figure 13) [18]. Instead, pyruvate treatment restored the cytotoxic effect of these chemotherapeutic agents in HT29-dx and HMM cells, suggesting that pyruvate action is required to restore the ROS-mediated drugs effects.

### 2.6. Pyruvate Restored Both Respiratory Activity and ATP Production in Both HT29-dx and HMM Cells

In contrast to normal differentiated cells, which primarily rely on mitochondrial oxidative phosphorylation to generate the energy needed for cellular processes, most cancer cells depend on aerobic glycolysis [8]. To investigate the involvement of respiratory chain as the electron flux in the pyruvate effect, we measured the complex I–III activity, the production of mitochondrial ATP and oxidized nicotinamide adenine dinucleotide/reduced nicotinamide adenine dinucleotide (NAD^+^/NADH) ratio with or without pyruvate supplementation.

**Figure 14:**
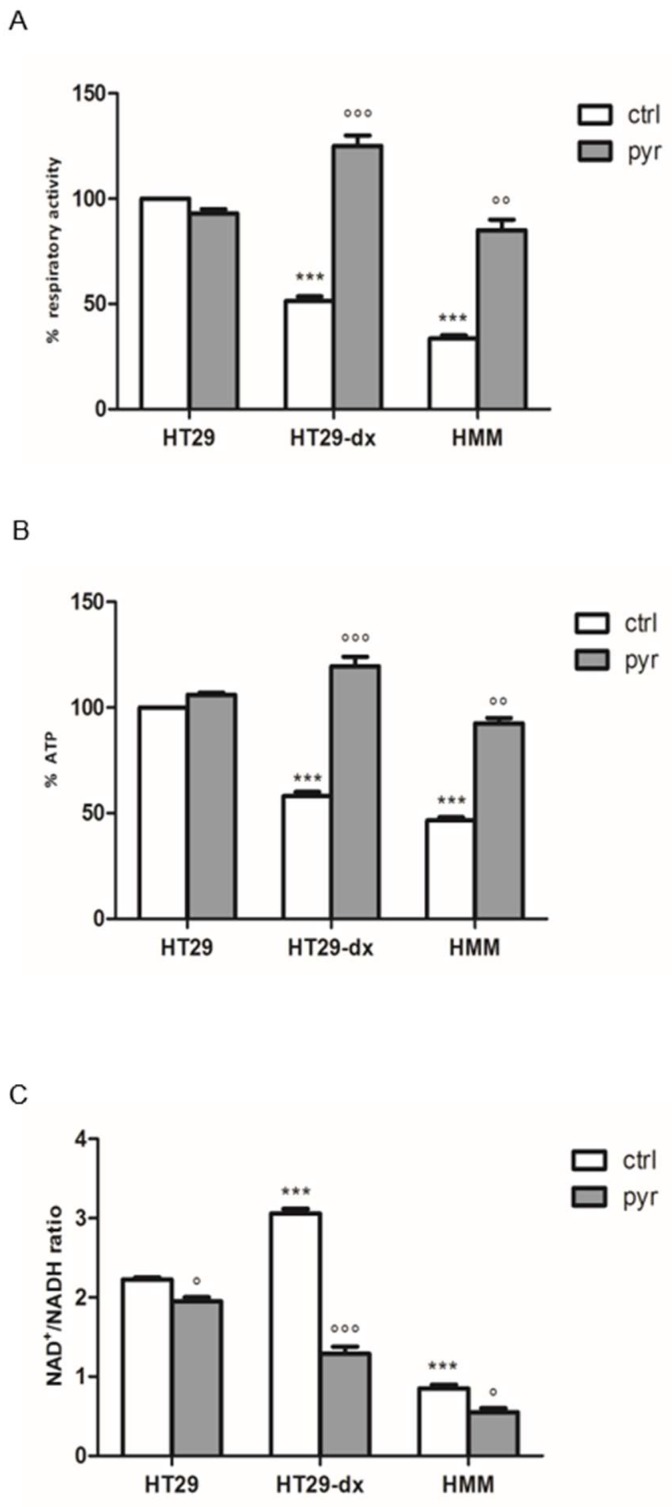
Analysis of respiratory activity and ATP synthesis with pyruvate in HT29, HT29-dx and HMM cells. (**A**) The cells were incubated in fresh medium in the absence or in the presence of pyruvate (pyr) 25 mM for 24 h. The cells were lysed, the mitochondrial fractions were isolated and the rate of reduction of cytochrome c was measured. (**B**) ATP was measured in triplicate in the mitochondrial extracts by a chemiluminescence-based assay. (**C**) intracellular NAD^+^/NADH ratio was measured in triplicate in the whole cells by a chemiluminescence-based assay. Measurements were performed in triplicate and data are presented as means ± SEM (*n* = 3). (**A**,**B**) HT29-dx and HMM cells in the absence of pyr versus HT29 and HMM cells in the absence of pyr (ctrl) *** *p* < 0.0001; HT29-dx cells in the presence of pyr versus HT29-dx cells in the absence (ctrl) of pyr °°° *p* < 0.0001; HMM cells in the presence of pyr versus HMM cells in the absence of pyr °° *p* < 0.001. (**C**) HT29 and HMM cells in the presence of pyr versus HT29-dx and HMM in the absence of pyr (ctrl) ° *p* < 0.01; HT29-dx and HMM cells in the absence of pyr versus HT29 cells in the absence (ctrl) of pyr *** *p* < 0.0001; HT29-dx and HMM cells in the presence of pyr versus HT29-dx and HMM cells in the absence (ctrl) of pyr °°° *p* < 0.0001 and ° *p* < 0.01.

We observed that HT29-dx and HMM cells had a significant lower activity of the respiratory chain and a significant reduced ATP level compared to HT29 cells (Figure 14A, B). The addition of pyruvate did not modify the respiratory activity and ATP production in HT29 cells. As shown in Figure 14, pyruvate was able to restore both respiratory activity and ATP synthesis in HT29-dx and HMM cells, at the same level of HT29 cells. In presence of pyruvate treatment, the intracellular NAD^+^/NADH ratio was lower in HT29-dx and HMM cells (Figure 14C). Thus, the cytosolic pyruvate might not be required as an exogenous electron acceptor to regenerate NAD^+^ via LDH reaction. These data support the idea that pyruvate supplementation might be essential as a carbon substrate for the synthesis of Krebs cycle intermediates and, consequently, for metabolic function of respiration.

### 2.7. A Functional Mitochondrial Respiratory Chain Was Crucial for the Reversion of MDR Phenotype in HT29-dx and HMM Cells

**Figure 15:**
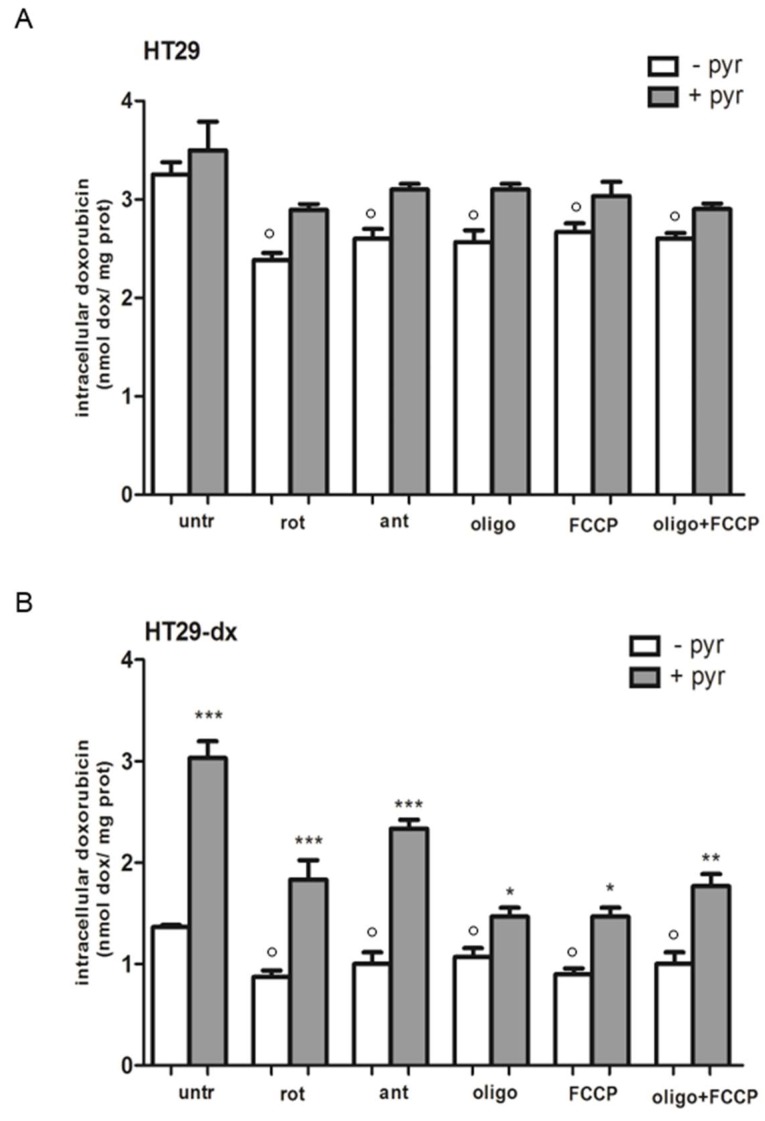

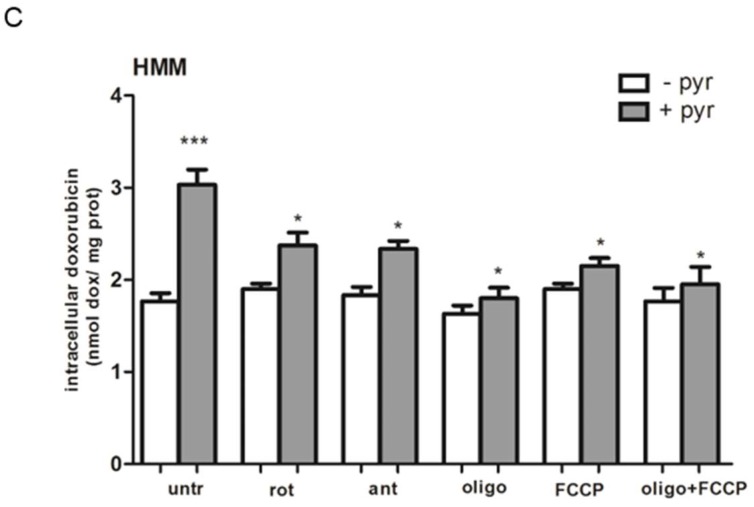
Doxorubicin accumulation with mitochondrial respiration inhibitors and with mitochondrial oxidative phosphorylation uncoupler in the absence or presence of pyruvate pre-treatment in: HT29 cells (**A**); HT29-dx cells (**B**); and HMM cells (**C**). The cells were incubated in fresh medium in the absence or in the presence of pyruvate (pyr) 25 mM for 4 h and then with rotenone (rot) 100 nM, antimycin (ant) 500 nM, oligomycin (oligo) 5 nM, 250 nM Carbonyl Cyanide-4-(Trifluoromethoxy) Phenylhydrazone (FCCP), or oligo plus FCCP for 24 h containing 5 µM doxorubicin for 24 h. The intracellular drug content was detected fluorometrically in duplicate (see Section 4 for details). The experiments were performed in triplicate and data are represented as mean ± SEM (*n* = 3). HT29-dx and HMM cells in the presence of pyr versus HT29-dx and HMM cells in the absence of pyr (untr) *** *p* < 0.0001; HT29-dx and HMM cells in the presence of inhibitors and pyr versus each inhibitor * *p* < 0.01; ** *p* < 0.001; *** *p* < 0.0001; HT29 and HT29-dx cells in the presence of inhibitors and in the absence of pyr versus untr ° *p* < 0.01

The intracellular doxorubicin accumulation was determined with or without pyruvate supplementation in the absence (untr) or in the presence of the mitochondrial respiration inhibitors rotenone (rot, inhibitor of Complex I), antimycin (ant, inhibitor of Complex III), oligomycin (oligo, inhibitor of Complex V), or a mitochondrial oxidative phosphorylation uncoupler Carbonyl Cyanide-4-(Trifluoromethoxy) Phenylhydrazone (FCCP), or oligo plus FCCP. When rotenone, antimycin, oligomycin, FCCP, or oligo plus FCCP were added to HT29, HT29-dx and HMM cells, we observed a decreased intracellular doxorubicin accumulation in HT29 and HT29-dx cells. In HMM cells, the mitochondrial inhibitors and uncoupler had no effect on doxorubicin accumulation, probably due to an existing reduction of activity of mitochondrial electron transport chain (ETC) in mesothelioma cells (Figure 15). As shown in Figure 6, pyruvate was able to significantly revert the doxorubicin accumulation in both HT29-dx and HMM cells. Moreover, pyruvate pre-treatment was able to prevent the reduced doxorubicin accumulation also in presence of mitochondrial inhibitors or with mitochondrial uncoupler, in both chemoresistant cells, suggesting a pivotal role of respiratory chain in the reversion of the MDR phenotype.

## 3. Discussion

To overcome the congenital or acquired chemoresistance, the development of new therapeutic strategies is becoming imperative and a better understanding of the mechanisms involved in this process is necessary.

All experiments were performed using the colon cancer HT29 cell line and its chemoresistant counterpart HT29-dx cell line, generated in our laboratory by culturing parental cells in the presence of increasing concentrations of doxorubicin for 20 passages, and the results obtained in HT29-dx cells were validated on the malignant mesothelioma highly chemoresistant HMM cell line. In the present work, we demonstrated that pyruvate treatment re-establishes the sensibility to different chemotherapeutic drugs in HT29-dx and HMM cells through the restoration of respiratory chain functionality.

Pyruvate constitutes a critical branch point in cellular carbon metabolism [19] and, as the lactate/pyruvate reaction is always at equilibrium [20], the metabolic fate of pyruvate determines whether glycolysis is followed by oxidative phosphorylation (OXPHOS) or by lactic fermentation [6].

Firstly, we demonstrated that HT29, HT29-dx and HMM cells had a different carbon metabolism. In particular, we reported that HT29-dx cells had a higher glucose consumption compared to HT29 cells, whereas HMM cells showed a lower glucose consumption compared to HT29 cells, accompanied by a lower production of pyruvate and, thus, a higher production of lactate without an increase in ^13^CO_2_ production. In addition, the HMM cells showed a significant decrease in ^13^CO_2_ production, suggesting a different fate of glucose carbon 2 and a reduced mitochondrial functioning. HT29-dx and HMM cells undergo lactic fermentation to produce anaerobic microenvironments. Indeed, high lactate concentrations and acidic pH, representative features of glycolytic tumors, have been associated with poor prognosis and a more aggressive phenotype [21].

Several metabolic alterations, driven by genetic and epigenetic factors, have been correlated to drug activity and clinical outcome, supporting the hypothesis that cancer metabolism is intimately linked to chemoresistance [22]. Particularly, several regulatory enzymes in glycolysis have been implicated in promoting a drug-resistant cancer phenotype.

Even if we demonstrated that glycolysis, assessed by both enzymatic analysis and ^13^C NMR technique, was not modified in HT29-dx cells compared to HT29 cells, the increased PCK2 mRNA levels in HT29-dx cells was in agreement with the metabolomic results, reflecting that a part of lactate is synthesized from the pyruvate through the PC and the PEPCK actions. These data are in accordance with results reported by Zhao et al., who, using prostate cancer patient database information, revealed that higher levels of PCK2 expression are associated with more aggressive tumors and lower survival rates [23].

Altered metabolism comprises a multifactorial process of concerted action of genes, proteins and metabolites that generate a characteristic cancer phenotype. However, nowadays, most studies have focused only on the few proteins involved in cancer metabolism and resistance towards anticancer drugs [24]. It is well known that LDH-A is involved in tumor initiation and cancer metabolism resulting in lactate production. We observed that the LDH-A mRNA expression and the LDH intracellular activity were significantly increased in HT29-dx cells compared to HT29 cells, in accordance with Zhou et al., who showed an association between an increased expression and the activity of LDH-A in paclitaxel-resistant breast cancer cells, underscoring that siRNA knockdown of LDH-A reverses taxol sensitivity in resistant cells [25].

Knowing that HT29-dx cells had a lower production of pyruvate, we restored their sensibility to doxorubicin and to rhodamine 123 after 24 h of treatment with pyruvate, increasing the drug-induced cytotoxic effect through the decrease of MRP1 activity without modifying its gene and protein expression.

Although we lack a mechanistic explanation of the impaired MRP1 activity after pyruvate treatment, our results strongly demonstrated a restoration in intracellular doxorubicin accumulation in chemoresistant cells after pyruvate incubation. We hypothesize that the increased pyruvate-induced ATP synthesis might be used in other numerous biological processes and for energy maintenance that were impaired in chemoresistant phenotype.

These data were confirmed on HMM cells. Some studies report that a lower level of mitochondrial pyruvate carrier-1 (MPC-1) protein expression is related to poor survival rate in diverse types of tumors including lung, colon, kidney clear cell, prostate and esophagus squamous cell carcinomas [26,27].

The evidence that UK5099 compound promotes a significant reduction in doxorubicin accumulation induced by pyruvate in HT29-dx and HMM cells, is also supported by Li et al. and by Zhong Y et al. Indeed, the authors demonstrated that *MPC-1* gene knockout cells or blocking MPC activity revealed a metabolic reprogramming to aerobic glycolysis with reduced pyruvate transport into mitochondria and ATP production, and, as a consequence, the cells became more migratory and resistant to both chemotherapy and radiotherapy [17] [16].

It is noteworthy that one of the cytotoxic effect of doxorubicin [28], of 5-fluorouracil [29] and of oxaliplatin [30] is the increase of ROS production and its reduction to semi-quinone radicals that generate O^2−^, H_2_O_2_ and OH. Then, the activation of nicotinamide adenine dinucleotide phosphate (NADPH) oxidase, and the genesis of redox cycles through iron catalyzed mechanisms and the complex I of the mitochondrial respiratory chain are also involved. The pyruvate treatment in association with different drugs decreased cells viability, increasing ROS production in both HT29-dx and HMM cells.

Recent evidence supports the hypothesis that acquired resistance to therapy is accompanied by a metabolic shift toward respiratory metabolism, reflecting that metabolic plasticity can have a role in survival of cells responsible for tumor relapse. Furthermore, cells lacking a functional mitochondrial electron transport chain (ETC) require pyruvate for proliferation, confirming that pyruvate auxotrophy accompanies loss of mitochondrial respiration and that pyruvate substitutes have essential metabolic function for respiration [31]. In our experimental conditions, in chemoresistant cells, supplementation of pyruvate could act as a carbon substrate for synthesis of biosynthetic intermediates that are normally involved in respiration for ATP synthesis. Indeed, sodium oxamate, enhancing the availability of pyruvate for mitochondrial metabolism, is able to reprogram cellular metabolism to obtain a sensitive phenotype. Furthermore, our results strongly suggest that intramitochondrial pyruvate can restore both respiratory activity and ATP synthesis of HT29-dx and HMM cells at the same level to the one of HT29 cells. We observed also that the HT29-dx and HMM cells accumulate more doxorubicin in the presence of pyruvate, suggesting that there is a link between the altered mitochondrial oxidative phosphorylation and drug resistance. Indeed, we demonstrated that ETC inhibitors and FCCP, when added to both HT29 and HT29-dx cells, decreased intracellular doxorubicin accumulation, whereas they had no effect on HMM cells where the respiratory chain was impaired. Moreover, in both HT29-dx and HMM cells, pyruvate is sufficient to restore the doxorubicin accumulation, when treatment with any of the inhibitors is used to block the respiratory chain activities, reinforcing the role of electron transport chain in the reversion of MDR phenotype.

In conclusion, we demonstrated that pyruvate treatment restores the respiratory chain functionality and diminishes MRP1 activity, improving the effectiveness of the chemotherapeutic agents in these resistant cancer cells.

## 4. Material and Methods

### 4.1. Cells and Reagents

Human colon adenocarcinoma (HT29) cell line obtained from the American Type Culture Collection (Rockville, MD, USA) was grown as a sub-confluent monolayer in Roswell Park Memorial Institute (RPMI) 1640 medium, containing 2 mM l-glutamine, 1% (*v*/*v*) antibiotic/antimycotic solution, and 10 % (*v*/*v*) fetal bovine serum (FBS).

The chemoresistant counterpart (HT29-dx cells) line was generated by culturing parental cells in the presence of increasing concentrations of doxorubicin for 20 passages [13]. HT29-dx cells have higher Pgp, MRP1 and breast cancer resistance protein (BCRP) than HT29 cells; moreover, compared to HT29 cells, HT29-dx cells have a higher IC_50_ for doxorubicin, irinotecan, oxaliplatin and 5-fluorouracil representing a reliable model of MDR cells [15]. For the present work, HT29-dx cells were grown in RPMI 1640 medium containing 150 nM doxorubicin.

The human malignant mesothelioma (HMM) cell line used in this study was established from the pleural effusion of patients with malignant mesothelioma (MM), collected from the Biologic Bank of Malignant Mesothelioma, S.S. Antonio and Biagio Hospital, Alessandria, Italy, where the histological characterization was performed. As reported in several cell lines derived from the pleural effusion of patients with MM, our HMM cells show a MDR phenotype [32], which is caused by an overexpression of Pgp and/or MRPs [33,34].

Unless otherwise specified, reagents were purchased from Sigma-Aldrich (Milan, Italy), whereas plastic ware was from Falcon (Becton Dickinson, Franklin Lakes, NJ, USA).

### 4.2. Metabolite Assays and ^13^C NMR Technique

After removing the perchloric acid (40%) and denaturing protein by centrifugation (3000× *g* for 5 min), the supernatant was neutralized with 20% KOH and used for metabolite determinations. Glucose utilization and product formation (glutamine, glutamate, alanine, pyruvate, lactate and CO_2_) were measured by enzymatic methods and NMR technique using 2-^13^C-glucose at T0 and T1 (after 48 h) time cultures [35,36,37]. The intramitochondrial transport of NADH was calculated by the following formula: 2 × glucose consumption − [production of lactate + 2 × 2-^13^C-glycerol production]. All the measurements were performed by Metabolys SAS (Lyon, France).

### 4.3. Glycolysis and Gluconeogenesis Array

Glycolysis and gluconeogenesis mRNA expression were analyzed using a PrimerPCR Pathway 96-well plate, and data were elaborated using Bio-Rad CFX Manager Software (3.1 version, Glycolysis and Gluconeogenesis H96, Human, Bio-Rad Laboratories, Hercules, CA, USA).

### 4.4. Quantitative Real-Time PCR (RT-PCR)

Total RNA was obtained with RiboZol RNA-Extraction Reagent. Two micrograms of total RNA were reversely transcribed into cDNA, in a final volume of 20 μL, using the iScript™ cDNA Synthesis Kit (Bio-Rad, Hercules, CA, USA) according to the manufacturer’s instructions. The RT-PCR was carried out using iTaq™ Universal SYBR^®^ Green Supermix (Bio-Rad), according to the manufacturer’s instructions in a final volume of 20 μL with specific primers for the quantitation of the genes of interest and the housekeeping gene (human beta-2 microglobulin).

The primers were: for *B2M*: 5′-AGCAAGGACTGGTCTTTCTAATCT-3′ (forward), 5′-ATGTCTCGATCCCACTTAACTATCC-3′ (reverse), for *MRP1*: 5′-CCTGTGATCCACCAGAAGGT-3′ (forward), 5′-TCTGGTCAGCCCAACTCTCT-3′ (reverse); and for *LDH-A*: 5′-AGCACTCTCAACCACCTGCT-3′ (forward), 5′-TGGGAGTTCACCCATTAAGC-3′ (reverse).

The relative quantification was performed by comparing each PCR product with the housekeeping PCR product using Bio-Rad software gene expression quantitation (Bio-Rad Laboratories).

### 4.5. Measurement of Lactate Dehydrogenase LDH Activity

Cells were seeded in a six-well tissue culture plate (500,000 cells/well) and then incubated for 24 h in fresh medium containing 5 µM doxorubicin.

Monolayer cells were washed twice with PBS, detached with scraper and resuspended in 200 µL of PBS and sonicated (1 bursts of 10 s, amplitude 40%; Hielscher UP200S ultrasound sonicator, GmbH, Teltow, Germany). Aliquots of 5 µL of cell lysate were supplemented with an 82.3 mM triethanolamine phosphate hydrochloride (TRAP, pH 7.6) reaction mixture containing 0.25 mM NADH and 0.5 mM piruvic acid for the measurement of LDH.

Intracellular enzyme activity, measured spectrophotometrically as absorbance variation at 340 nm (37 °C), was expressed as nmol NADH oxidized/min/mg proteins.

### 4.6. Doxorubicin Accumulation

The cells were seeded in a six-well tissue culture plate (500,000 cells/well). After 24 h, the cells were incubated in fresh medium in the absence or in the presence of 75 mM oxamate for 24 h, of 20 μM UK5099 (MCP, mitochondria pyruvate carrier inhibitor) for 48 h, of 100 nM rotenone (rot, inhibitor of Complex I), of 500 nM antimycin (ant, inhibitor of Complex III), of 5 nM oligomycin (oligo, inhibitor of Complex V) and of 250 nM mitochondrial oxidative phosphorylation uncoupler Carbonyl Cyanide-4-Trifluoromethoxy Phenylhydrazone (FCCP) for 24 h. The cells were washed twice in PBS, scraped and centrifuged at 13,000 rpm for 5 min at 4 °C. Cell pellets were resuspended in 600 µL of a 1:1 mixture of ethanol/0.3 N HCl and sonicated (1 bursts of 10 s, amplitude 40%; Hielscher UP200S ultrasound sonicator, GmbH, Teltow, Germany). The protein content of cells was assessed with the bicinchoninic acid protein assay kit. The amount of intracellular doxorubicin was detected using a PerkinElmer LS-5 spectrofluorimeter. Excitation and emission wavelengths were 485 and 590 nm, respectively. A blank was prepared in the absence of cells in each set of experiments and its fluorescence was subtracted from that measured in each sample. Fluorescence was converted into nmol doxorubicin/mg cell proteins using a calibration curve prepared previously.

### 4.7. Rhodamine 123 Accumulation

The cells were grown up to confluence in six-well culture plate and incubated in the absence or presence of 25 mM pyruvate for 24 h. Cells were washed with PBS, detached with cell dissociation solution (Sigma-Aldrich), centrifuged at 13,000 rpm for 5 min and re-suspended in 0.5 mL culture medium containing 5% *v*/*v* FBS. A 100 μL aliquot was taken, sonicated and used for the measurement of the protein content. Rhodamine 123 (1 ng/mL) was added to the remaining sample. After 30 min at 37 °C, cells were washed three times with PBS and re-suspended in 500 μL PBS. The intracellular fluorescence of rhodamine 123 (index of Pgp/ABCB1 and MRP1/ABCC1 activity) was measured fluorometrically using a LS-5 spectrofluorometer (PerkinElmer, Waltham, MA, USA). Excitation and emission wavelengths were 480 and 530 nm for rhodamine 123. Fluorescence was expressed as RFU/mg cell proteins.

### 4.8. Western Blot Analysis

Membrane proteins (45 μg/lane) were obtained with the Pierce Cell Surface Protein Isolation Kit (Pierce Biotechnology, Thermo Scientific) and protein extracts were subjected to SDS-PAGE and then transferred to PVDF membrane (Immobilon-P, Millipore, Bedford, MA, USA). Due to difficulty finding a non-modulated housekeeping membrane protein in resistant tumor cells, it was used Ponceau stain. PVDF membrane was blocked in 5% *w*/*v* non-fat dry milk, 0.1% tween-20, Tris-buffered saline (TBS) over day at room temperature and probed with the following antibodies, diluted in 2% *w*/*v* non-fat dry milk 0.1% tween-20 TBS: anti-MRP1 mouse monoclonal antibody (diluted 1:250, Cruz Biotechnology, Santa Cruz, CA, USA). After overnight incubation at 4 °C, the membrane was washed with 0.1% *v*/*v* tween-20 TBS for 30 min and subjected for 1 h at room temperature to a horseradish peroxidase-conjugated anti-mouse (diluted 1:3000, Bio-Rad Laboratories). The membrane was washed again with 0.1% *v*/*v* tween-20 TBS for 30 min and proteins were detected and quantified by ChemiDoc™ MP System (Bio-Rad Laboratories). Densitometric analysis was carried out using Image J software (http://rsbweb.nih.gov/ij/).

### 4.9. Cell Viability

The neutral red uptake assay provides a quantitative estimation of the number of viable cells in a cellular culture. Cells were seeded in 96-well tissue culture plates (25,000 cells/well). After 24 h, the cells were incubated in fresh medium in absence or presence of pyruvate 25 mM containing doxorubicin (doxo 5 µM), 5-fluorouracil (5-FU 150 µM) and oxaliplatin (OPT 30 µM) for 24 and 48 h.

The plate was then incubated for 1 h at 37 °C with a medium containing Neutral Red solution. The cells were subsequently washed and rinsed with the stop buffer (1:1 of 4.02 g trisodium citrate in 153 mL H_2_O, 0.8 mL HCl 0.1 N in 86 mL H_2_O and 25 mL of 95% *v*/*v* methanol). The absorbance was read at 540 nm and the cell viability was assessed by measuring the percentage of cells stained with neutral red dye. The viability of untreated cells was considered 100%; the results were expressed as percentage of viable cells in each experimental condition versus untreated cells.

### 4.10. Measurement of Total Cellular ROS Production

Cells were seeded in six-well tissue culture plates (500,000 cells/well). After 24 h, the cells were incubated in fresh medium in absence or presence of pyruvate 25 mM containing 5 µM doxorubicin (for 24 h). The cells were washed twice in PBS and detached with scraper and centrifuged at 13,000 rpm for 5 min at 4 °C. Cell pellets were resuspended in a final volume of 0.5 mL PBS, incubated for 30 min at 37 °C with 10 μM of the fluorescent probe 5-(and-6)-chloromethyl-2′,7′-dichlorodihydro-fluorescein diacetate-acetoxymethyl ester (DCFDA-AM), centrifuged at 13,000× *g* at 37 °C, washed twice with PBS and re-suspended in 0.5 mL PBS. The fluorescence of each sample, proportional to the amount of ROS, was read at 504 nm (λ excitation) and 529 nm (λ emission), using a Packard EL340 microplate reader (Bio-Tek Instruments, Winooski, VT, USA). The results were expressed as RFU/mg cell proteins.

### 4.11. Isolation of Mitochondria and Measurement of Complex I–III Activity

To isolate mitochondrial fractions, 5 × 10^6^ cells were washed twice in PBS, then lysed in 0.5 mL buffer A (50 mM Tris, 100 mM KCl, 5 mM MgCl_2_, 1.8 mM ATP, 1 mM EDTA, pH 7.2), supplemented with protease inhibitor cocktail III (Sigma Aldrich, Milan, Italy), 1 mM PMSF and 250 mM sodium fluoride (NaF). Samples were sonicated (1 bursts of 10 s, amplitude 40%), clarified by centrifuging at 2000 rpm for 1 min at 4 °C, and the supernatant was collected and centrifuged at 13,000 rpm for 5 min at 4 °C. This supernatant was discarded and the pellet containing mitochondria was washed in 0.5 mL buffer A and resuspended in 0.25 mL buffer B (250 mM sucrose, 15 mM K_2_HPO_4_, 2 mM MgCl_2_, 0.5 mM EDTA, 5% *w*/*v* BSA).

A 20 µL aliquot was sonicated and used for the measurement of protein content.

The activity of Complex I–III was measured on 50 µL of non-sonicated mitochondrial samples, resuspended in 150 µL buffer C (5 mM K_2_HPO_4_, 5 mM MgCl_2_, 5% *w*/*v* BSA) and after 1 min of incubation 100 µL buffer D (25% *w*/*v* saponin, 50 mM K_2_HPO_4_, 5 mM MgCl_2_, 5% *w*/*v* BSA, 0.12 mM cytochrome c-oxidized form) was added for 7 min at room temperature.

The reaction was started with 0.15 mM NADH and the absorbance was read at 550 nm by a Lambda 3 spectrophotometer (PerkinElmer). Under these experimental conditions, the rate of cytochrome c reduction, expressed as nmol cyt c reduced·min^−1^·(mg cell protein)^−1^, was dependent on the activity of both Complex I and Complex III.

### 4.12. ATP Assay

The amount of mitochondrial ATP was measured with the ATP Bioluminescent Assay Kit (FL-AA, Sigma-Aldrich), using a Synergy HT Multi-Mode Microplate Reader (Bio-Tek). The ATP amount was quantified as relative light units (RLU) and data were converted into nmol ATP/mg mitochondrial proteins.

### 4.13. NAD^+^/NADH Assay

NAD^+^ and NADH were measured using the NAD^+^/NADH Quantification Colorimetric Kit from Sigma-Aldrich (Milan, Italy), which allows determination of NAD^+^, NADH and their ratio, without the requirement to purification steps. The intracellular NAD^+^/NADH assay was performed according to the manufacturer’s specifications; NAD^+^ and NADH levels were adjusted for protein contents.

### 4.14. Measurement of Mitochondrial Pyruvate Concentration

Cells were harvested at 80% confluent and 4.5 × 10^7^ cells were left for mitochondria isolation, according to the mitochondria/cytosol fractionation method (Section 4.11). Fifty microliters of lysis buffer, containing 1% PMSF in RIPA buffer, were added to the isolated mitochondria and homogenized by sonication. Mitochondrial pyruvate concentration was determined by pyruvate enzymatic assay, as described by Marbach E, et al. and Van der Bossche D, et al. [38,39].

### 4.15. Statistical Analysis

Measurements were performed in triplicate. All data in text and figures are provided as means ± SEM. The results were analyzed by a one-way Analysis of Variance (ANOVA) and Tukey’s test. *p* < 0.05 was considered significant.

## Supplementary Materials

Supplementary materials can be found at http://www.mdpi.com/1422-0067/19/11/3550/s1.

## Abbreviations

HT29	human colon adenocarcinoma cell line
HT29-dx	human chemoresistant counterpart colon adenocarcinoma cell line
HMM	human malignant mesothelioma cell line
MDR	multidrug resistance
ROS	reactive oxygen species
MRP1	multidrug resistance-associated protein 1
PCK2	phosphoenolpyruvate carboxykinase 2
LDH	lactate dehydrogenase
FBP1	fructose-1,6-bisphosphatase 1
SLC2A4	solute carrier family 2, member 4
