# Supplementary material for: Pyruvate Treatment Restores the Effectiveness of Chemotherapeutic Agents in Human Colon Adenocarcinoma and Pleural Mesothelioma Cells
Source: Int J Mol Sci. 2018 Nov 10;19(11):3550. doi: 10.3390/ijms19113550 (PMC6274794; doi:10.3390/ijms19113550)
Supplement: Supplementary file 1 [file ijms-19-03550-s001.pdf]

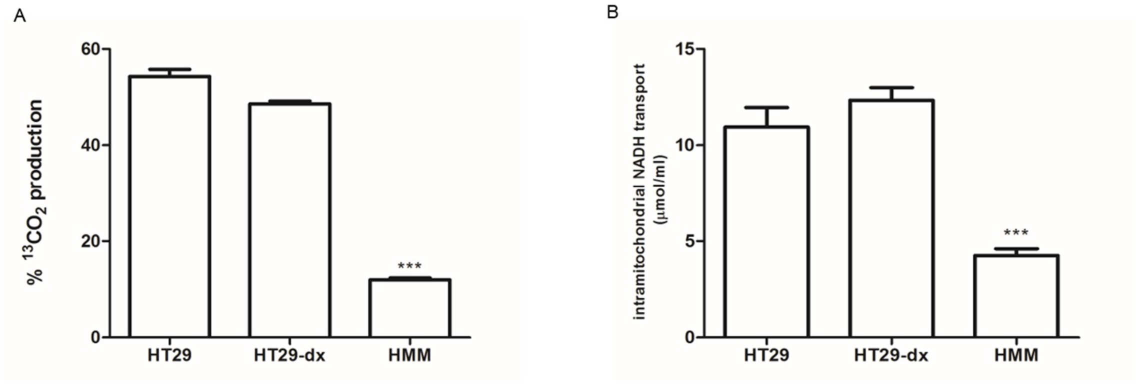

Figure S1. CO_2 formation (**A**) and intramitochondrial NADH transport (**B**) levels in HT29, HT29-dx and HMM cancer cells. Results in quadruplicate are presented as means ± SEM ($n = 4$), HMM versus HT29: *** $p < 0.0001$.
